# Sudden sensorineural hearing loss in patients with vestibular schwannoma

**DOI:** 10.1038/s41598-020-80366-2

**Published:** 2021-01-21

**Authors:** Koichiro Wasano, Naoki Oishi, Masaru Noguchi, Ko Hentona, Seiichi Shinden, Tsubasa Kitama, Nobuyoshi Tsuzuki, Taiji Kawasaki, Yoshihiko Hiraga, Yasuhiko Takei, Kaoru Ogawa

**Affiliations:** 1 Division of Hearing and Balance Research, National Institute of Sensory Organs, National Hospital Organization Tokyo Medical Center, 2-5-1 Higashigaoka, Meguro, Tokyo, 152-8902 Japan; 2 Department of Otolaryngology, National Hospital Organization Tokyo Medical Center, Tokyo, Japan; 3 Department of Otolaryngology-Head and Neck Surgery, Keio University School of Medicine, 35 Shinanomachi, Shinjuku, Tokyo, 160-8582 Japan; 4 Department of Otolaryngology, Hino Municipal Hospital, Tokyo, Japan; 5 Department of Otolaryngology, Saiseikai Utsunomiya Hospital, Tochigi, Japan; 6 Department of Otolaryngology, Hiratsuka City Hospital, Kanagawa, Japan; 7 Department of Otolaryngology, Japanese Red Cross Shizuoka Hospital, Shizuoka, Japan; 8 Department of Otolaryngology, Inagi Municipal Hospital, Tokyo, Japan

**Keywords:** Neurology, Epidemiology

## Abstract

Clinical features of sudden sensorineural hearing loss (SSNHL) associated with vestibular schwannoma (VS) are not fully understood. Determining a treatment plan and explaining it to patients requires clinicians to clearly understand the clinical features related to the tumor, including SSNHL. To identify the full range of clinical features of VS-associated SSNHL, especially recovery of hearing following multiple episodes of SSNHL and what factors predict recovery and recurrence. A multicenter retrospective chart review was conducted in seven tertiary care hospitals between April 1, 2011, and March 31, 2020. We collected and analyzed dose of administered steroid, pure-tone audiometry results, and brain MRIs of patients diagnosed with VS-associated SSNHL. Seventy-seven patients were included. They experienced 109 episodes of audiogram-confirmed SSNHL. The highest proportion of complete recoveries occurred in patients with U-shaped audiograms. The recovery rates for the first, second, and third and subsequent episodes of SSNHL were 53.5%, 28.0%, and 9.1%, respectively. Recovery rate decreased significantly with increasing number of SSNHL episodes (*P* =0 .0011; Cochran-Armitage test). After the first episode of SSNHL, the recurrence-free rate was 69.9% over 1 year and 57.7% over 2 years; the median recurrence time was 32 months. Logarithmic approximation revealed that there is a 25% probability that SSNHL would recur within a year. SSNHL in patients with VS is likely to recur within one year in 25% of cases. Also, recovery rate decreases as a patient experiences increasing episodes of SSNHL.

## Introduction

Sporadic vestibular schwannoma (VS) accounts for approximately 80% of cerebellopontine angle tumors^1^. An estimated 12 to 26% of patients diagnosed with VS develop sudden sensorineural hearing loss (SSNHL)^2,3^. Sporadic VSs are often treated with corticosteroids according to treatment protocols used for idiopathic SSNHL^4–6^. Although SSNHL is a common symptom of VS, little information exists about the extent to which this symptom resolves and about its recurrence rate. Also, previous studies have generally had few available patients for study^4–6^ and some patients with VS-associated SSNHL have one or more additional episodes after initial hearing recovery^7^.

From published reports, it is difficult to determine the detailed clinical characteristics of VS-associated SSNHL, the efficacy of a particular plan of treatment, and the long-term status and progression of hearing loss and recovery, as only a small number of cases were analyzed in these studies. Regardless, the detailed clinical features of the first episode of VS-associated SSNHL sufferers has not been sufficiently studied. Other questions also arise: Does recovery rate of VS-associated SSNHL change over time? Is recovery rate related to tumor grade? Is recovery rate related to dosage of steroids administered? Answers to these questions are important, as the indication for surgery of VSs remains controversial^8^. We hypothesized that the detailed clinical features of VS associated SSNHL would contribute to the decision of surgeons and even patients.

In the present study, we conducted a retrospective chart review of a relatively large sample of VS patients with SSNHL. Seven general tertiary-level hospitals in Japan contributed data to the analysis, with the goal of clarifying the detailed clinical features of VS-associated SSNHL and of determining the recovery rate and recurrence rate.

## Methods

We conducted a retrospective chart review of the patients who developed SSNHL between April 1, 2011, and March 31, 2020, on April 2020 as part of the Keio Academical Otolaryngology Research Unit (KAORU) Project. Participating hospitals in this project are Keio University, National Hospital Organization Tokyo Medical Center, Saiseikai Utsunomiya Hospital, Hiratsuka City Hospital, Japanese Red Cross Shizuoka Hospital, Hino Municipal Hospital, and Inagi Municipal Hospital. This study was approved by the institutional review boards of Keio University School of Medicine Ethics Committee (approval number 2020-0033), National Hospital Organization Tokyo Medical Center (approval number R20-046), Saiseikai Utsunomiya Hospital (approval number 2020-19), Japanese Red Cross Shizuoka Hospital (approval number 2020-15), Inagi Municipal Hospital (dated 23/06/2020), Hiratsuka City Hospital (approval number 02-003), and Hino Municipal Hospital IRB (dated 24/03/2021). The requirement of written informed consent was waived because of the study’s retrospective design. We defined SSNHL as follows: “The patient is aware of sudden hearing loss, and the patient’s hearing thresholds measured through audiometry testing at 0.25, 0.5, 1, 2, or 4 kHz are increased in one or more frequencies by more than 20 dB.” All candidate patients for this study were being treated with corticosteroids.

We included two kinds of patients presenting with SSNHL: (1) cases of SSNHL in the patients with *known* VS, and (2) cases of SSNHL that were identified first, followed soon after by a diagnosis of VS. All candidate participants had accompanying audiograms; we excluded cases in which onset of SSNHL was not verified by pure-tone audiometry testing. From every included patient, we gathered and analyzed their chart data on the dose of oral or intravenous steroid administered, their audiogram results, their MRI images related to the VS diagnosis, and the number and timing of recovery and recurrences.

The severity classification of SSNHL was defined based on criteria of the Japan Ministry of Health, Labor and Welfare research group^9^. According to these criteria, hearing severity is categorized into four grades based on hearing thresholds measured with pure-tone audiometry at 0.25, 0.5, 1, 2, and 4 kHz: Grade 1, mean hearing level (averaged across 0.25, 0.5, 1, 2, and 4 kHz) is < 40 dB; Grade 2, mean hearing level is ≥ 40 dB but < 60 dB; Grade 3, mean hearing level is ≥ 60 dB but < 90 dB; and Grade 4, mean hearing level is ≥ 90 dB^9^.

Our criteria for judging treatment outcome (i.e., amount of hearing improvement) was also based on the criteria of the Japan Ministry of Health, Labor and Welfare research group for measuring hearing improvement after recovering from SSNHL^9^; there were some minor modifications done for this study. First, the mean hearing threshold of each patient was calculated based on thresholds measured at five test frequencies (0.25, 0.5, 1, 2, and 4 kHz). We used these results to categorize hearing improvement as “complete recovery,” “marked improvement,” “partial improvement,” or “no change”^9^. The patient’s hearing was considered to be completely recovered for the following three types of audiogram results: (1) if his/her mean hearing threshold was ≤ 20 dB; (2) in cases where hearing threshold on the affected side before onset was known, the mean hearing threshold recovered to within 10 dB of their pre-onset hearing level; (3) in cases where the mean hearing thresholds of the affected and unaffected sides were equivalent before onset, the hearing threshold of the affected side recovered to within 10 dB of the healthy side’s hearing level; (4) in cases with dip form, the hearing threshold of affected frequency recovered to within 10dB of the pre-onset or healthy side's hearing level. A patient was considered to have marked improvement in hearing when the average of the hearing levels measured at the five frequencies improved by ≥ 30 dB. A patient was considered to have partial improvement in hearing when the average hearing level was ≥ 10 dB but < 30 dB. A patient’s hearing outcome was designated as no change when the average hearing improved < 10 dB. Treatment outcome was assessed one or more months after onset of SSNHL by performing audiograms, and a judgment was made using the audiogram after there was no further improvement. We defined recovery rate as the percentage of patients who achieved complete recovery.

Since we were interested in whether the configuration of hearing loss (i.e., form or shape of the audiogram) could be a prognostics indicator of recovery rate or recurrence^10^, we also evaluated the shape of each patient’s audiograms. The audiogram shapes were defined according to the following characteristics.

Reference points **A–G** in audiogram forms 1–7:

 Hearing threshold assessed at 0.125 kHz: **A**

 Hearing threshold assessed at 0.25 kHz: **B**

 Hearing threshold assessed at 0.5 kHz: **C**

 Hearing threshold assessed at 1 kHz: **D**

 Hearing threshold assessed at 2 kHz: **E**

 Hearing threshold assessed at 4 kHz: **F**

 Hearing threshold assessed at 8 kHz: **G**

1. Low-frequency, ascending form:
  [(**A** + **B** + **C**) ÷ 3] – [(**D** + **E** + **F** + **G**) ÷ 4] > 15 dB.
2. U-shaped form:
  (Poorest in **C**, **D**, and **E**) - (Poorest in **A** and **B**) > 15 dB.
  and (Poorest in **C**, **D**, and **E**) - (Poorest in **F** and **G**) > 15 dB.
3. High-frequency descending form:
  [(**F** + **G**) ÷ 2] – [(**D** + **E**) ÷ 2] > 15 dB.
4. Flat form:
  The difference between [(**B** + **C**) ÷ 2], [(**D** + **E**) ÷ 2], and [(**F** + **G**) ÷ 2] is < 15 dB.
5. Profound form:
  Hearing thresholds are at ceiling levels at 3 or more frequencies in **B**, **C**, **D**, **E**, and **F.**
6. Dip form:
  Does not meet any of the above criteria,
  and (Poorest in **B**, **C**, **D**, **E**, and **F**) - (all other frequencies) > 20 dB.
7. Other form:
  The shape of the audiogram did not fit any of the six shapes above.

We used the Cochran–Armitage trend test (CATT) to compare the recovery rate of the first, second, and subsequent episodes of SSNHL^11,12^. The CATT is a standard statistical procedure used to analyze factors in a complex disease that are measured at the categorical level^13^. Besides recovery rates, we also used the CATT to determine whether the high-grade severity class of HL was related to a poorer prognosis. We used Chi-square tests to statistically evaluate the recovery rate of each audiogram shape.

Using MRI, we examined whether the severity of first episodes of SSNHL correlated with the size of the VS tumor in the internal auditory canal (IAC), as graded by the Koos classification system^14^. Spearman’s rank correlation test was used to evaluate differences. One-way ANOVA was used to analyze the relationship between the audiogram shape of the first episode of SSNHL with the Koos tumor size classification. Fisher's exact test was used to evaluate the relationship between the VS tumor in the IAC fundus and the shape of the audiogram obtained during the first episode of SSNHL and the recovery rate of the first SSNHL episode. Fisher’s exact test was used to analyze the relationship between tumor growth and recovery rate. Tumor growth was defined as an increase of 2 mm or more in the tumor’s maximum diameter axis.

After the first episode of SSNHL, we quantified how long the patient was recurrence-free (i.e., period of good hearing) using Kaplan–Meier survival curves. A recurrence included both occurrence of SSNHL relapse and gradual progression of hearing loss after initial recovery which was judged to have occurred if a patient’s hearing threshold increased by 20 dB or more (i.e., average hearing level obtained at five frequencies) above their hearing level measured at the time of recovery of his/her first episode of SSNHL. A patient’s data were censored in the Kaplan–Meier analyses if the patient underwent a subsequent intervention (e.g., surgery and/or radiation treatment), if the patient dropped out of the study, or if the patient did not develop hearing loss before the last day of this study, March 31, 2020. To approximate data for the Kaplan–Meier curve in the prior 48 months, we used a logarithmic model in the following form:

$${\text{Y(recurrence free)}} = {\text{K}}^{{\text{X(year)}}}$$

The percentage of patients who had recurrences every year was determined.

We adopted the Kaplan–Meier method and log-rank test to examine whether the recurrence of hearing loss was associated with tumor growth, whether the tumor was in the fundus of the IAC, and the recovery of the first episode of SSNHL.

We used GraphPad Prism 9.0.0 for Windows, GraphPad Software, San Diego, California USA, www.graphpad.com to approximate the logarithmic model and create bar and pie charts, and EZR ver.1.40 for the other statistical analyses^15^. *P* < 0.05 was considered significant. The Strengthening the Reporting of Observational Studies in Epidemiology (STROBE) statement was followed^16^.

## Results

Seventy-seven patients (32 males, 45 females) met the inclusion criteria (Table 1). Mean (standard deviation [SD]) age at onset of SSNHL was 48.9 (14.2) yr; median (interquartile range [IQR]) age was 49 (19) yr. Of the 77 patients included in this study, 43 had a VS tumor on the right side, and 34 had a tumor on the left side (Table 1). There were a total of 107 episodes of SSNHL experienced. Of these, 71 were first SSNHL episodes, 25 were second SSNHL episodes, and 11 were third and subsequent SSNHL episodes. Thirty-nine patients had single episode, 22 patients had 2 episodes, and 7 patients had 3 or more episodes. And 9 patients had gradual progression of hearing loss after SSNHL. The mean (SD) initial dose of prednisolone administered was 49.4 (26.4) mg/day.

**Table 1:** Clinical data of the patients.

Age at onset of sudden sensorineural hearing loss [years old]	
Mean (standard deviation)	48.9 (14.2)
Median (interquartile range)	49 (19)
Male:Female	32:45
Right:Left	43:34
Grade 1:2:3:4 (First sudden sensorineural hearing loss)	42:17:10:4
The mean (standard deviation) initial dose of prednisolone administered [mg/day]	49.4 (26.4)
Koos classification, grade 1:2:3:4	39:27:9:2
Tumor occupation in the fundus of IAC yes:no	22:55
Tumor growth yes:no	22:31

**Figure 1:**
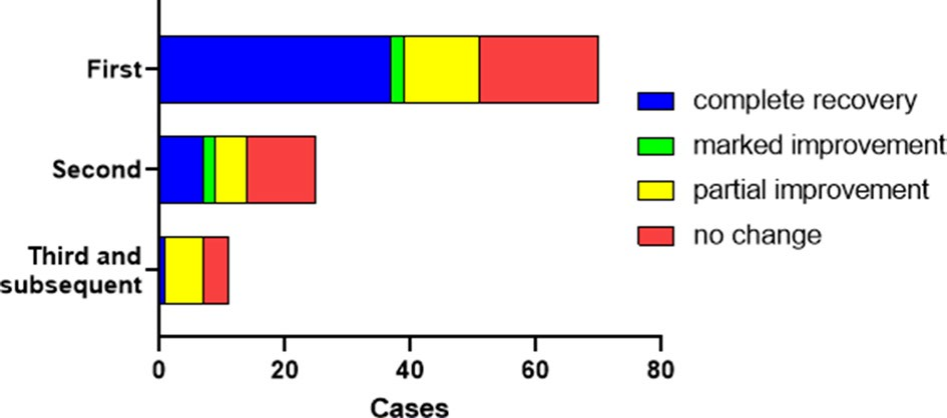
Hearing outcomes for successive episodes of sudden sensorineural hearing loss. The recovery rate decreased significantly as the number of hearing loss episodes increased, as evaluated by the Cochran-Armitage trend test (*P* = 0.0011). A hearing outcome was designated as “no change” when the average hearing threshold improved by < 10 dB. This bar chart was created using GraphPad Prism 9.0.0 for Windows, GraphPad Software, San Diego, California USA, www.graphpad.com.

We were able to evaluate the amount of recovery for 71 first SSNHL episodes (Fig. 1). For first SSNHL episodes, 38 (53.5%) showed complete recovery of hearing; 2 (2.8%) showed marked improvement; 12 (16.9%) showed partial improvement; and 19 (26.7%) showed no change. For the 25 second SSNHL episodes, 7 (28.0%) showed complete recovery; 2 (8.0%) showed marked improvement; 5 (20.0%) showed partial improvement; and 11 (44.0%) showed no change. For the 11 third and subsequent SSNHL episodes, 1 (9.1%) showed complete recovery; 6 (54.5%) showed partial improvement; and 4 (36.4%) showed no change. The CATT revealed that the recovery rate decreased with each successive hearing loss episode (*P* = 0.0011).

**Figure 2:**
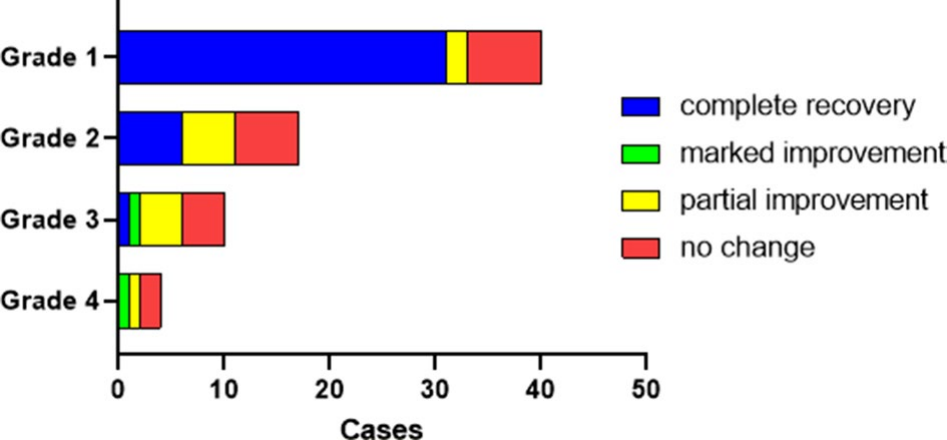
Frequency distribution of severity of hearing loss (Grade 1–4) in the first sudden sensorineural hearing loss episode and outcomes of each grade (n = 71 episodes). The degree of recovery (complete recovery, marked improvement, partial improvement, or no change) decreased with increasing hearing-loss severity, a trend that was significant as revealed by the Cochran-Armitage trend test (*P* = 0.0000020). This bar chart was created using GraphPad Prism 9.0.0 for Windows, GraphPad Software, San Diego, California USA, www.graphpad.com.

The severity of HL for the first SSNHL episodes, stratified by classification of recovery (complete to no change) is shown in Fig. 2. The amount of recovery decreased as HL severity increased (Grade 1 to 4), a trend that was significant (*P* < 0.01; CATT). That is, there were progressively fewer instances of complete recovery in those classified as Grade 4 (most severe) compared to Grade 3, Grade 2, and Grade 1 (least severe). The greatest proportion of complete recovery classifications occurred in those with Grade 1 HL, while no complete recoveries occurred in those with Grade 4 HL (Fig. 2).

**Figure 3:**
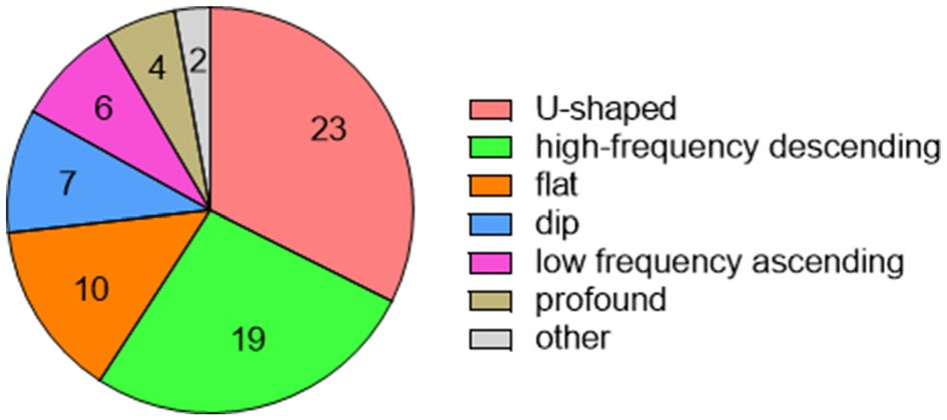
Distribution of patients’ audiogram forms obtained during the first episode of sudden sensorineural hearing loss. “Other” means that the audiogram characteristics did not adequately fit the descriptions of any of the six defined forms (see Methods). This pie chart was created using GraphPad Prism 9.0.0 for Windows, GraphPad Software, San Diego, California USA, www.graphpad.com.

**Figure 4:**
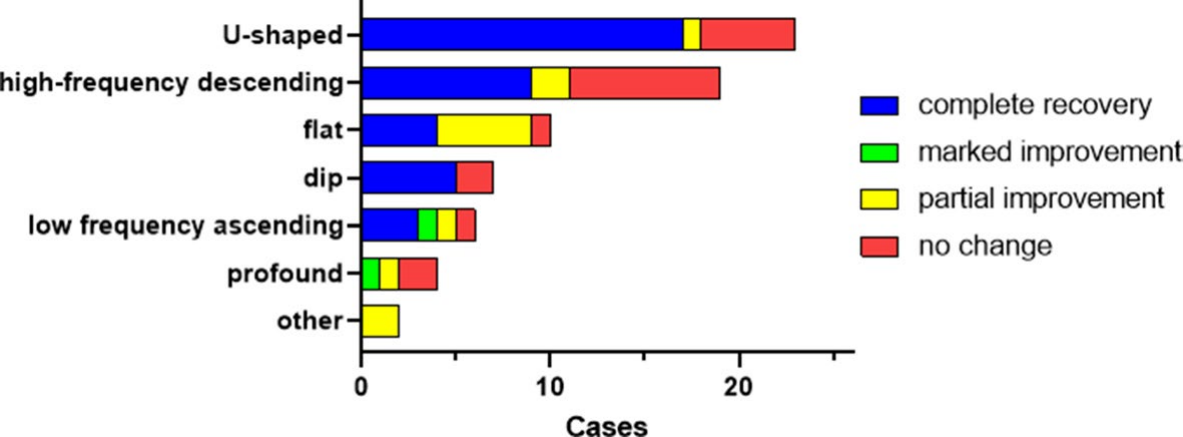
Frequency distribution of audiogram forms in the first sudden sensorineural hearing loss episode, stratified by degree of recovery. Audiogram shape was significantly related to recovery rate (*P* = 0.048). Residual analysis based on the results of Chi-squared test revealed recovery of hearing in patients having U-shaped audiograms was significantly greater than in patients having the other audiogram forms (*P* < 0.05) and the amount of recovery for the profound form was significantly less than that of the other audiogram forms (*P* < 0.05). This bar chart was created using GraphPad Prism 9.0.0 for Windows, GraphPad Software, San Diego, California USA, www.graphpad.com.

Most audiogram shapes (59%) collected during the first SSNHL episodes had high-frequency descending forms or U-shaped forms (Fig. 3). Audiogram shape was significantly related to recovery rate (*P* =0 .048) (Fig. 4). Residual analysis based on the results of Chi-squared test revealed recovery of hearing in patients having U-shaped audiograms was significantly greater than in patients having the other audiogram forms (*P* < 0.05) and the amount of recovery for the profound form was significantly less than that of the other audiogram forms (*P* < 0.05) (Fig. 4).

We also evaluated any possible relationships between VS-tumor size and severity of hearing loss, tumor size in the IAC, and audiogram shape (data not shown). Koos classification of tumor size and severity of hearing loss were unrelated (*r* =  − 0.116, *P* =0 .327; Spearman's rank correlation test). There was also no significant correlation between tumor size in the IAC and severity of hearing loss (*r* =  − 0.14, *P* = 0.24; Spearman's rank correlation test). Similarly, there was no significant relationship between Koos classification and audiogram shape (*P* = 0.93) or tumor size in the IAC and audiogram shape (*P* = 0.81; one-way ANOVA). The presence of a VS tumor in the fundus of the IAC had no significant relationship with the audiogram shape for first SSNHL episodes (*P* = 0.70) or amount of recovery shown in the first SSNHL episodes (*P* = 0.60; Fisher’s exact test). Finally, tumor growth (yes/no) showed no significant relationship with the recovery of hearing loss (*P* = 0.78; Fisher’s exact test).

**Figure 5:**
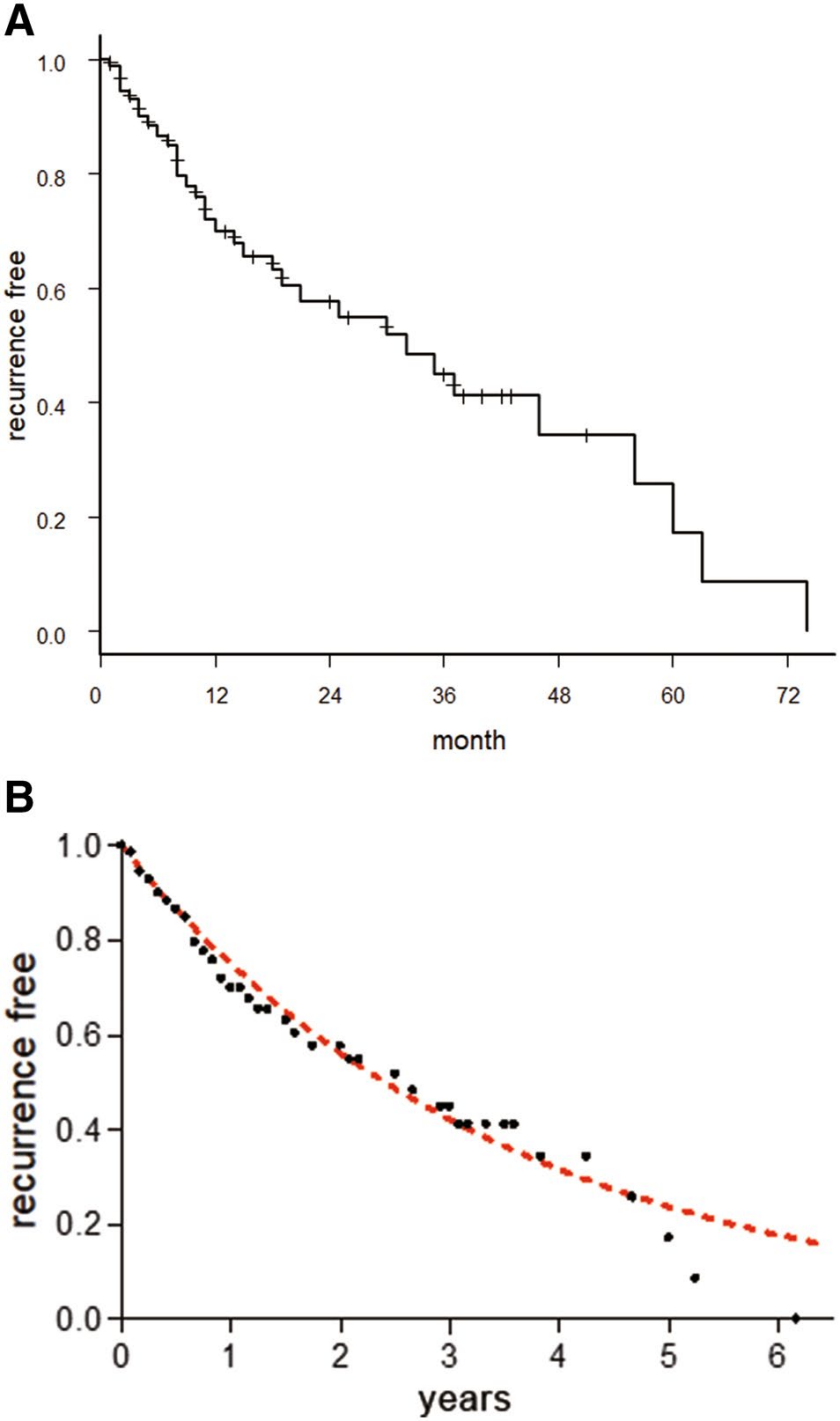
(**A**) Kaplan–Meier curve of recurrence data showing recurrence-free time after the first sudden sensorineural hearing loss episode. The recurrence-free (i.e., period of good hearing) rate was 69.9% over one year and 57.7% over two years; the median recurrence time for a subsequent sudden sensorineural hearing loss episode was 32 months. We used EZR ver.1.40 ^15^ to create Kaplan–Meier curve. (**B**) Probability of recurrence over time. Logarithmic approximation (orange squares) of Kaplan–Meier graph for recurrence of sudden sensorineural hearing loss plotted along with the recurrence free rate (filled circles). The best fit value of K was .75, indicating that there is a 25% probability of sensorineural hearing loss recurrence every year. We used GraphPad Prism 9.0.0 for Windows, GraphPad Software, San Diego, California USA, www.graphpad.com to approximate the logarithmic model.

**Figure 6:**
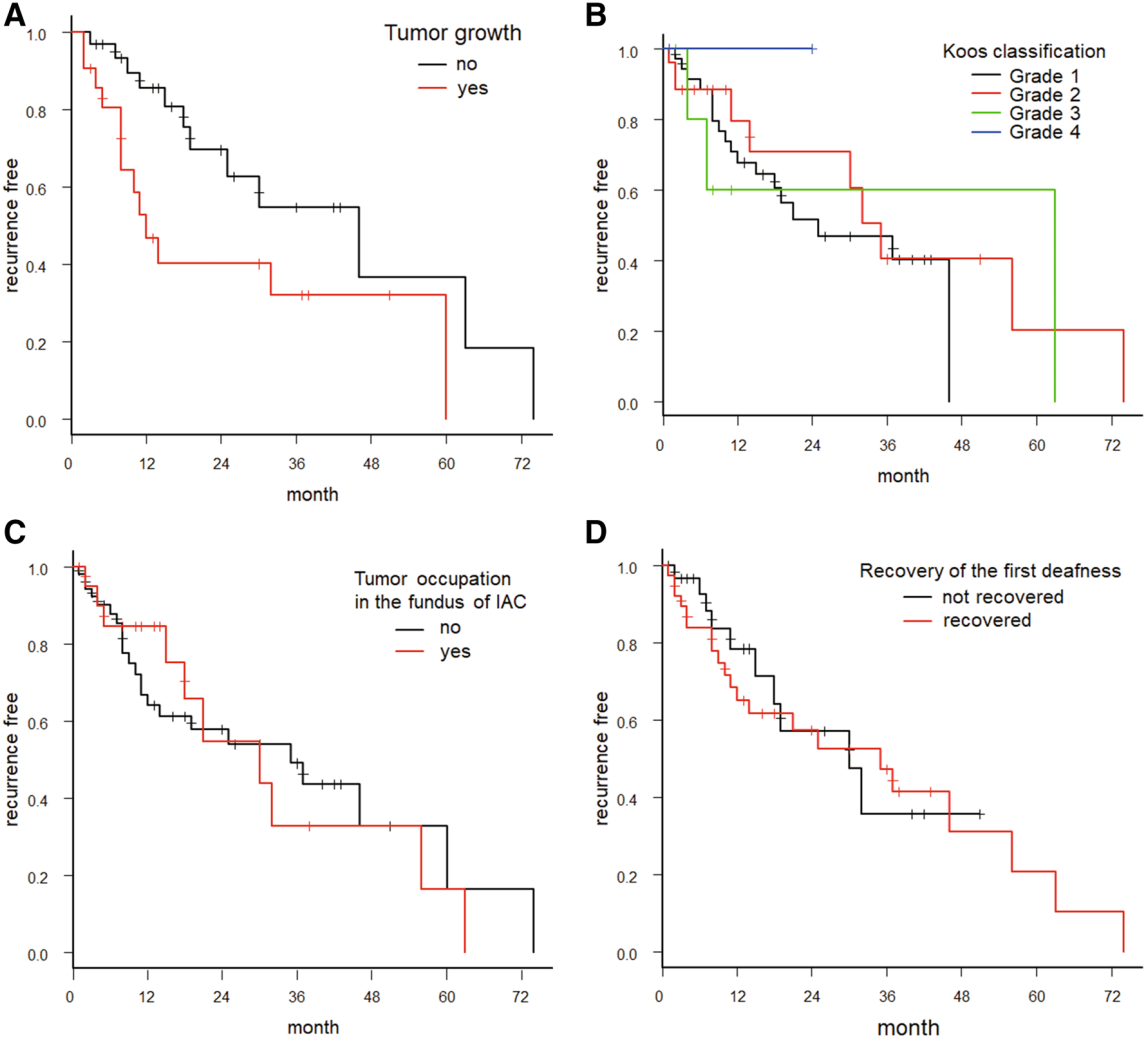
Kaplan–Meier curves for recurrence of sensorineural hearing loss and whether VS tumor showed growth (increase of 2 mm or more in the tumor’s maximum diameter axis). Median months (95% confidence interval); NA: not available. (**A**) Kaplan–Meier curves for recurrence-free period plotted separately for tumor growth (yes, red) or not (no, black). Tumor growth (no), 46 months (19-NA); Tumor growth (yes), 12 months (8-NA); *P* = 0.030. (**B**) The Kaplan–Meier curves for recurrence-free period plotted separately by Koos size classification of tumor. Grade 1, 25 months (12-NA); Grade 2, 35 months (14-NA); Grade 3, 63 months (4-NA); Grade 4, NA; *p* = 0.82. (**C**) The Kaplan–Meier curves for recurrence-free period plotted separately for VS tumors in the fundus of internal auditory canal (IAC) or not. Occupying the fundus (no, black), 35 months (12–60); Occupation (yes, red), 30 months (15–56); *P* = 0.92. (**D**) Kaplan–Meier curves for recurrence-free period plotted separately for recovered from first sudden sensorineural hearing loss episodes, or not. Not recovered, 30 months (12-NA); Recovered, 35 months (11–56); *P* = 0.73. We used EZR ver.1.40 ^15^ to create these Kaplan–Meier curve.

After the first episode of SSNHL, the recurrence-free rate was 69.9% over one year and 57.7% over two years (Fig. 5A). The median (95% confidence interval [CI]) recurrence time for another SSNHL episode was 32 (18–56) months. By performing a logarithmic approximation on the graph created with the Kaplan–Meier method, the best-fit value for K was 0.75 (95% CI, 0.74–0.76; *r*^2^ = 0.98). This result means that there is a 25% probability of SSNHL recurrence every year (Fig. 5B). The Kaplan–Meier method and log rank test revealed that the recurrence of SSNHL was significantly related to tumor growth (*P* = 0.030) (Fig. 6A), but not to Koos classification (*P* = 0.82) (Fig. 6B), tumor occupation in the fundus of IAC (*P* = 0.92) (Fig. 6C), or recovery of the first episode of hearing loss (*P* = 0.73) (Fig. 6D).

## Discussion

This study reports on the largest number of VS-associated SSNHL cases to date. There have been several earlier reports showing that hearing recovers with corticosteroid treatment in patients diagnosed with VS^4–6,17–19^. However, our study is the first report to elucidate the detailed clinical features of VS-associated SSNHL, in particular, recurrence of SSNHL over time, clinical course of subsequent episodes of SSNHL, audiogram configuration, and tumor characteristics to recovery of hearing after SSNHL.

Prior to the present study, the largest survey on idiopathic SSNHL in Japan had 3194 cases^20^. This study did not specifically evaluate only cases of SSNHL with MRI-confirmed VS. Although the survey was a different kind of study, we used the same criteria as those investigators did to evaluate hearing recovery after SSNHL. This allowed us to compare more directly our results with theirs.

In that survey, the recovery rate was 41.2% (1317/3194), while in our study the recovery rate for the first episode of SSNHL was 53.5% (38/71). Although the recovery rate in our study was not markedly different from that in the survey on idiopathic SSNHL, it did tend to be higher than that in the survey study (*P* = 0.051; Fisher’s exact test). However, our study and the survey study appeared to diverge some in the age of the study subjects. In the present study, the mean age [SD] at onset of the first episode of SSNHL was significantly younger than that in the survey on idiopathic SSNHL (48.9 [14.2] years vs. 54.2 years [17.0]; *P* = 0.0018; Welch's test), and the highest proportion of patients in the survey study was in the 60–69 year age range^20^.

Recovery of hearing in patients with idiopathic sudden hearing loss has been associated with the shape of the patient’s audiogram^21^. One study reported that 10.9% (90/828) of patients with idiopathic SSNHL exhibit trough-shaped audiograms (i.e., U-shaped form and dip shaped form in the present study), a shape that predicts likely hearing recovery^22^. In the present study, 41.1% (30/73) of patients exhibited the equivalent of the trough-type audiograms; this was a significantly higher percentage than that in the aforementioned report (*P* < 0.01; Fisher’s exact test). Taken together, these data indicate that otolaryngologists need to consider VS screening mainly for relatively young patients, even if their hearing loss completely recovers. Recovery of hearing is highly probable, especially in patients that exhibit U-shaped form and dip-shaped form of audiograms.

Since we found no clear relationships between MRI findings and hearing-related clinical course in VS patients—except for a modest connection (*P* = 0.030) between VS tumor growth and recurrence of hearing loss—it is important to continuously assess hearing levels after the first presentation of SSNHL. In addition, repeated imaging in these patients should be judiciously conducted in order to detect possible explanations for clinical changes in patients that might be related to VS growth. A similar conclusion was reached by Lee et al.^23^.

Kaplan–Meier survival analyses were useful in understanding the detailed clinical features of VS-associated SSNHL. Our logarithmic approximation analyses indicated that in our cohort, SSNHL recurred at a rate of 25% per year and that the recovery rate for the second and subsequent episodes of hearing loss was significantly lower than that for the first episode. Therefore, in VS patients choosing the treatment option of “wait and scan,” it is necessary to inform those with SSNHL that hearing loss may recur. As the success rate of hearing-preservation surgery is improving with recent improvements in surgical procedures and advances in intraoperative hearing monitoring^24,25^, surgery in these patients might be favorably considered before recurrence of hearing loss, even if the patient’s first episode of SSNHL has resolved completely.

Although the present study was the largest to date on VS-associated SSNHL cases and the first to elucidate the pattern of recurrence of SSNHL in this patient population, it has some limitations. First, this was a retrospective study, and as such, not all patients who presented with SSNHL underwent imaging tests. In Japan, MRIs are often performed on patients whose hearing loss has not recovered, not on patients who present with no hearing loss. So, there is a possibility that these latter kinds of patients, who have not been imaged, may indeed have a VS. Therefore, the actual recovery rate of SSNHL caused by VS may be higher. Second, although speech audiometry is commonly used as a follow-up test for VS^26^, we could not assess speech audiometry data in this study. Since speech audiometry is not routinely performed during the follow-up of individuals having a differential diagnosis of idiopathic SSNHL, we were unable to collect enough speech audiometry data for analysis.

## Conclusion

We found that the recovery rate of VS-associated SSNHL decreases with increasing episodes of hearing loss. This, together with our finding that SSNHL recur within one year in 25% of cases, should prompt otolaryngologists and neurosurgeons to follow up on the hearing status of VS patients in addition to monitoring VS status with MRI. Alternatively, as the success rate of hearing-preservation surgery has improved greatly in recent years, VS patients choosing to wait and scan might consider undergoing surgery before hearing loss recurs.

## Data Availability

The data that support the findings of this study are available from the corresponding author upon reasonable request.
