# Author Correction: Sudden sensorineural hearing loss in patients with vestibular schwannoma

**DOI:** 10.1038/s41598-022-18674-y

**Published:** 2022-08-22

**Authors:** Koichiro Wasano, Naoki Oishi, Masaru Noguchi, Ko Hentona, Seiichi Shinden, Tsubasa Kitama, Nobuyoshi Tsuzuki, Taiji Kawasaki, Yoshihiko Hiraga, Yasuhiko Takei, Kaoru Ogawa

**Affiliations:** 1 Division of Hearing and Balance Research, National Institute of Sensory Organs, National Hospital Organization Tokyo Medical Center, 2‑5‑1 Higashigaoka, Meguro, Tokyo, 152‑8902 Japan; 2 Department of Otolaryngology, National Hospital Organization Tokyo Medical Center, Tokyo, Japan; 3 Department of Otolaryngology‑Head and Neck Surgery, Keio University School of Medicine, 35 Shinanomachi, Shinjuku, Tokyo, 160‑8582 Japan; 4 Department of Otolaryngology, Hino Municipal Hospital, Tokyo, Japan; 5 Department of Otolaryngology, Saiseikai Utsunomiya Hospital, Tochigi, Japan; 6 Department of Otolaryngology, Hiratsuka City Hospital, Kanagawa, Japan; 7 Department of Otolaryngology, Japanese Red Cross Shizuoka Hospital, Shizuoka, Japan; 8 Department of Otolaryngology, Inagi Municipal Hospital, Tokyo, Japan

Correction to: *Scientific Reports* https://doi.org/10.1038/s41598-020-80366-2, published online 21 January 2021

The original version of this Article contained an error in the Methods section, regarding ethical approvals. The data of three patients included from Hino hospital were not covered by the original ethical approval, which read:

“This study was approved by the institutional review board, and the requirement of written informed consent was waived because of the study’s retrospective design (Keio University School of Medicine Ethics Committee approval number 2020-0033).”

These patients provided their consent separately and an additional ethical approval was issued by the IRB of Hino Municipal Hospital. Additional information on the approval for study execution by the other participating centres was also included in the corrected version. The approval statement now reads:

“This study was approved by the institutional review boards of Keio University School of Medicine Ethics Committee (approval number 2020-0033), National Hospital Organization Tokyo Medical Center (approval number R20-046), Saiseikai Utsunomiya Hospital (approval number 2020-19), Japanese Red Cross Shizuoka Hospital (approval number 2020-15), Inagi Municipal Hospital (dated 23/06/2020), Hiratsuka City Hospital (approval number 02-003), and Hino Municipal Hospital IRB (dated 24/03/2021). The requirement of written informed consent was waived because of the study’s retrospective design.”

The original Article has been corrected.

